# Recalcitrant Pyoderma Gangrenosum of the Face: A Case Report

**DOI:** 10.7759/cureus.57136

**Published:** 2024-03-28

**Authors:** Megan Jiang, Grace Zhang, Tsung-yen Hsieh

**Affiliations:** 1 Otolaryngology, University of Cincinnati College of Medicine, Cincinnati, USA; 2 Otolaryngology, University of Cincinnati Medical Center, Cincinnati, USA

**Keywords:** pyoderma gangrenosum surgical, head and neck pyoderma gangrenosum, head and neck, skin graft, pyoderma gangrenosum post surgery, facial pyoderma gangrenosum

## Abstract

Pyoderma gangrenosum (PG) is a rare autoinflammatory neutrophilic dermatosis. The ulcerative subtype presents with a tender nodule or pustule that progresses into a painful, necrotic ulcer.New lesions arise after minor trauma in one-third of patients, a phenomenon termed “pathergy.” We present a 62-year-old Caucasian female with primary sclerosing cholangitis, hepatic cirrhosis, chronic hepatitis B, and severe PG. At the initial presentation, she had lesions on her face and four extremities. She had severe full-thickness ulcerations on the bilateral cheeks and underwent incision and drainage with washout of bilateral maxillary abscesses, left sinus curettage, and wound debridement. She has required multiple hospitalizations for severe flares. Treatment with steroids was complicated by spinal compression fractures. Steroid-sparring agents were ineffective. Her lesions involved bilateral cheeks, temples, temporal scalp, and eyelids with oroantral fistulae. Her facial ulcerations included a large septal perforation causing saddle nose deformity and eradication of a branch of the left facial nerve causing incomplete eye closure. She underwent bilateral facial wound irrigation with antibiotic irrigation and wound debridement. Due to social factors, she has been lost to follow-up and a definitive etiology of her PG has not yet been elucidated. Although rare, PG should remain a consideration in patients with ulcerative lesions on the head and neck. Wound debridement is typically discouraged given the risk of pathergy, but there may be a role for surgical intervention in adequately immunosuppressed patients.

## Introduction

Pyoderma gangrenosum (PG) is a rare autoinflammatory neutrophilic dermatosis encompassing five distinct clinical subtypes [1]. Among these, ulcerative PG is the most prevalent, presenting with a tender nodule or pustule that rapidly progresses into a painful, necrotic ulcer [2]. Typically affecting individuals 25-54 years old, its occurrence shows little gender bias [3]. Notably, approximately one-third of patients experience the emergence of new lesions after minor trauma. This post-traumatic exaggeration in skin injury is termed “pathergy,” highlighting heightened skin vulnerability [1]. Although PG can manifest in isolation, up to 75% of cases coincide with systemic diseases, predominantly inflammatory bowel disease (IBD), inflammatory arthritis, and hematological disorders [1,4]. Moreover, PG lesions demonstrate a strong predilection for the lower extremities, particularly the pretibial region, with only 3%-5% of cases involving the head and neck [4,5]. Consequently, encounters with PG in the field of otolaryngology are exceedingly rare. This article was previously presented as a meeting abstract at the 2024 Triological Combined Sections Meeting on January 25-27, 2024.

## Case presentation

**Figure 1:**
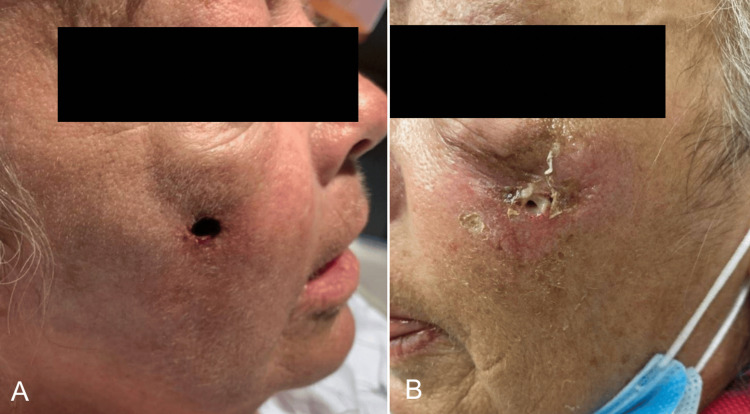
Initial Presentation Photographs at the initial presentation demonstrating the patient's bilateral facial pyoderma gangrenosum. A) Right side and B) left side.

Our case focuses on a 62-year-old Caucasian woman with a past medical history including primary sclerosing cholangitis (PSC), hepatic cirrhosis, and chronic hepatitis B infection. She is a well-known patient at our institution due to her chronic and severe PG of the face, complicated by recurrent superinfections (Figures 1A, 1B). She maintained a regimen of chronic systemic steroid therapy. Initially diagnosed with PG in February 2020 at an outside hospital, following wounds on her left lower extremity from minor trauma that failed to heal with conservative management, she later presented to our institution in July 2020 with new PG lesions spreading to her face, all four extremities, including the mons pubis, necessitating treatment with oral steroids.

**Figure 2:**
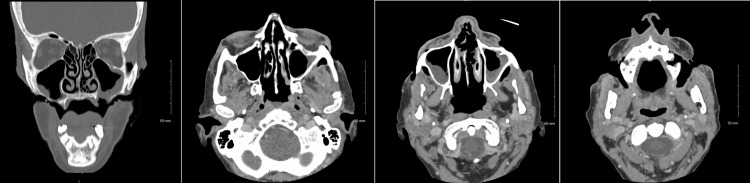
De-identified Patient's CT Imaging Coronal and transverse CT imaging of patient's disease.

Her condition notably featured severe full-thickness ulcerations on both cheeks, creating open defects that exposed her oral cavity. Computed tomography (CT) scan demonstrated disease extension into the masticator space, left-sided sinonasal involvement, and bilateral oroantral fistulae (Figure 2). MRI imaging was not completed as she had MRI-incompatible hardware implanted in her body. Interventions immediately following her presentation included incision and drainage, bilateral maxillary abscess washout, left sinus curettage, and facial wound debridement. Biopsies of facial lesions and buccal mucosa exhibited nonspecific acute and chronic inflammation and necrosis, with tissue culture positive for actinomyces, leading to treatment with vancomycin, piperacillin-tazobactam, and later amoxicillin-clavulanic acid with concurrent high-dose prednisone (60 mg).

**Figure 3:**
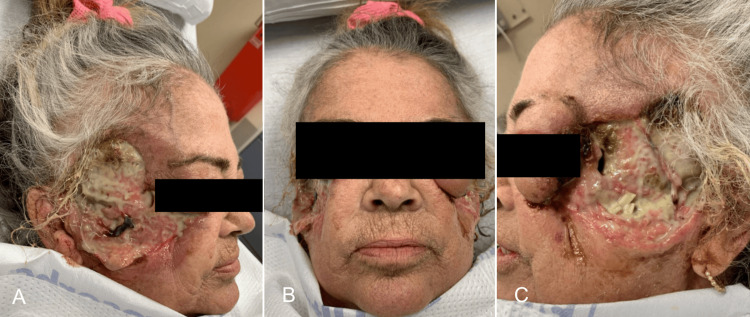
Disease Progression Through 2022 Patient PG progression in early 2022. PG: pyoderma gangrenosum. A) Right view, B) center, and C) left view.

Despite aggressive initial treatment, she was readmitted for PG flare with facial ulcerations several centimeters in size, angulated and irregularly shaped with gunmetal gray borders, copious drainage, and irregular borders. She necessitated a pulse dose of Solumedrol therapy that was effective, allowing her to be discharged on oral prednisone. Subsequent management involved infliximab injections in October 2020; however, wound cultures in September 2021 were positive for actinomyces and methicillin-resistant *Staphylococcus aureus* (MRSA), necessitating doxycycline. With her history of chronic actinomyces, Bactrim prophylaxis was started during another flare in November. Later admissions in December 2021 and March 2022, the latter requiring three days of intravenous immunoglobulin (IVIG), exemplified the complexity of her condition, complicated by positive MRSA cultures, spinal compression fractures from chronic steroid use, and hepatitis B reactivation (Figures 3A-3C). Entecavir treatment for hepatitis B was initiated alongside steroid-sparing agents.

**Figure 4:**
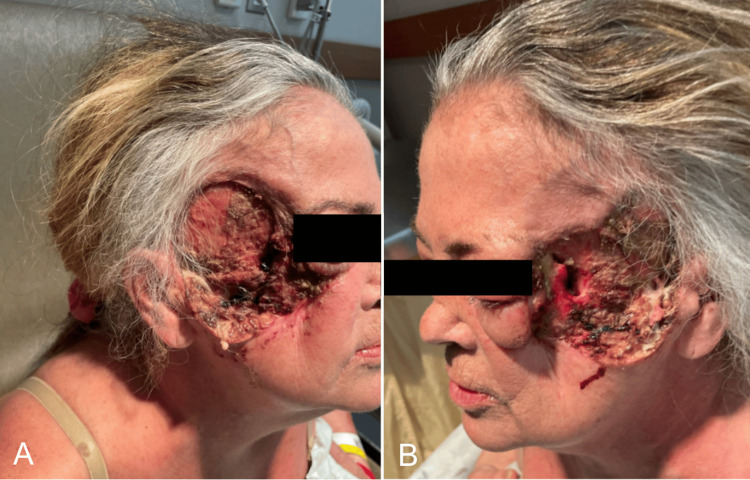
Patient Immediately Before Operation Patient's progressive pyoderma gangrenosum prior to her operation by ENT. A) Right side and B) left side.

Despite efforts to taper her steroid therapies, her PG progressed, revealing positive wound cultures in April 2022 for MRSA and *Klebsiella pneumoniae*. Colchicine, antibiotics, cyclosporine, and Solumedrol were titrated to address her acute infection. A repeat biopsy of the left cheek at this time showed dense neutrophilic infiltrate with necrosis and adjacent multinucleated cells and plasma cells. In June 2022, her course was complicated by yet another MRSA superinfection, aggravating her PG which now involved bilateral cheeks, temples, temporal scalp, and eyelids with complicating oroantral fistulae. Her facial ulcerations were severe and included a large septal perforation causing saddle nose deformity and eradication of both the zygomatic and frontal branches of the left facial nerve causing incomplete eye closure and significant ocular pains secondary to exposure keratopathy as she was lost to follow-up (Figures 4A, 4B).

**Figure 5:**
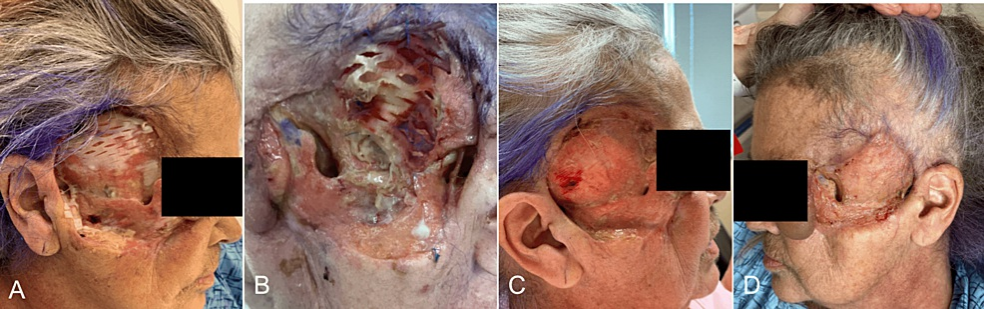
Patient After Operation Patient's pyoderma gangrenosum after her operation. (A, B) Right and left sides, respectively, with skin substitute immediately after her operation. (C, D) Right and left sides with healthy granulation tissue growing at later follow-up appointment.

Due to the rapidly evolving nature of the disease process, despite maximum medical management, surgical intervention became necessary. It was crucial to address the condition promptly to prevent further complications. Given the nature of the situation and the potential risks associated with pyoderma gangrenosum, all procedures were performed with utmost care to minimize adverse outcomes. We delicately performed bilateral facial wound irrigation, wound debridement, and application of Primatrix skin substitutes (Integra, New Jersey, USA). Skin substitute was utilized on culture-negative tissue to prevent a new wound from developing from the donor site. While autologous tissue may have been preferable due to decreased inflammation and contracture, a skin substitute was utilized instead to mitigate potential problems of widening the wound with a local flap or creating a new lesion at another site with a free flap. ACell powder (Integra, New Jersey, USA) was then applied to deep areas of the wound where the skin substitute did not contact. Bacitracin ointment was applied to Promatrix, and petroleum dressing was applied over (Figures 5A-5D) to protect the area.

**Figure 6:**
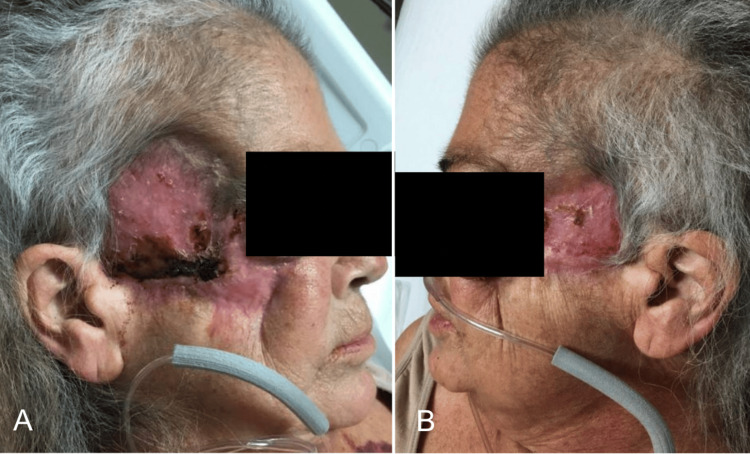
Patient at Six-Month Follow-Up Patient at six-month follow-up taking a combination of prednisone and infliximab. A) Right side and B) left side.

**Figure 7:**
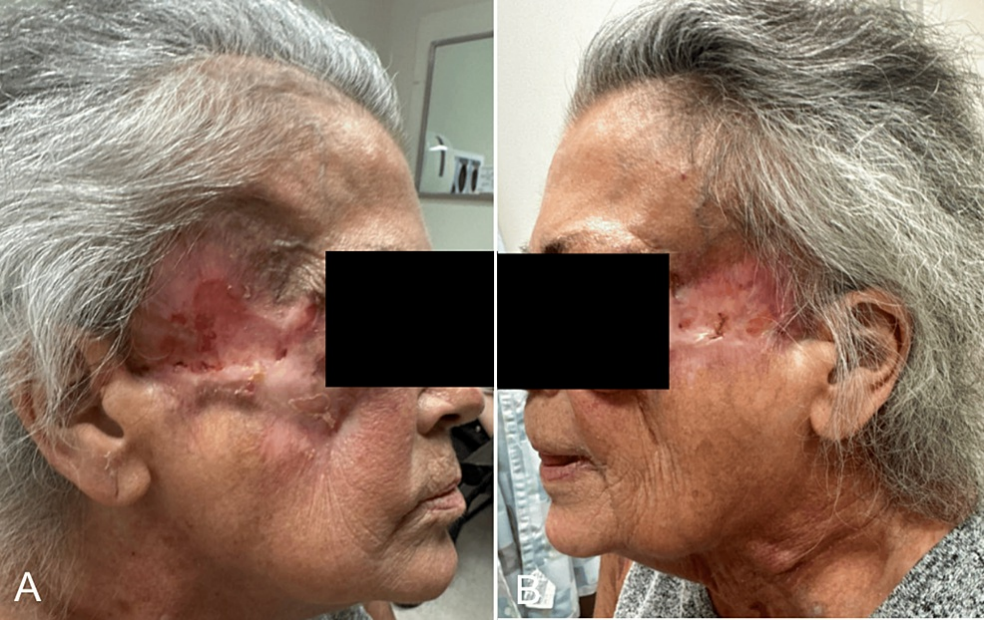
Patient at One-Year Follow-Up Patient at one-year follow-up taking prednisone and Bactrim prophylaxis. A) Right side and B) left side.

**Figure 8:**
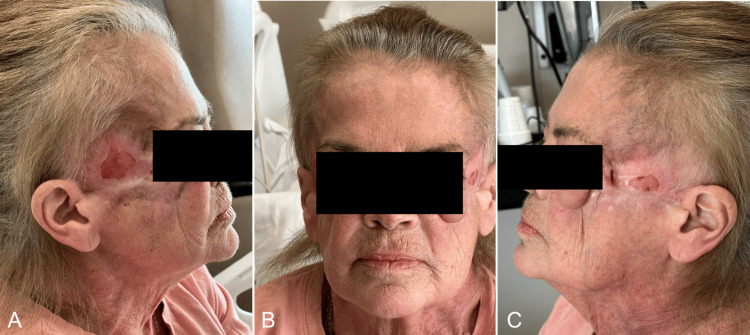
Patient at 1.5-Year Follow-Up Patient at her 1.5-year follow-up at the ENT outpatient clinic. A) Right side, B) center, and C) left side.

Post-surgery, a combination of prednisone and infliximab proved effective, although her irregular follow-ups at infusion appointments hindered consistent treatment. She continued to have occasional admissions for flares, culminating in a December 2023 admission for shortness of breath, revealing an orocutaneous fistula tracking from her upper left alveolus to her right temple (Figures 6A, 6B). She was discharged on prednisone and Bactrim prophylaxis, with dermatology and our outpatient follow-up (Figures 7A, 7B, 8A-8C). This approach aimed to address both the acute and long-term aspects of her condition, emphasizing the importance of multidisciplinary care in managing complex cases like hers. While the definitive etiology of her PG remains elusive, her history of primary sclerosing cholangitis (PSC) and positive antinuclear antibody (ANA) testing at a 1:150 homogeneous titer suggests autoimmune involvement. Interestingly, her anti-Sjogren's syndrome A (anti-SSA) and anti-Sjogren's syndrome B (anti-SSB) testing were negative. Colonoscopy ruled out IBD, although challenges in socioeconomic follow-up have hindered a comprehensive PG work-up.

## Discussion

Diagnosing PG poses a significant challenge due to its diverse subtypes and nonspecific histologic and laboratory findings [2]. Typically considered a diagnosis of exclusion, a thorough work-up is necessary to rule out other potential etiologies, particularly infectious or vascular [1]. The head and neck represent atypical sites for PG lesions, which further complicates the diagnostic process in this presentation. A retrospective study of 219 cases of head and neck pyoderma gangrenosum (HN PG) found that dermatologists correctly diagnosed PG in 58.1% of cases, while non-dermatologists made the correct diagnosis in 33.3% of cases. Frequently, PG was mistaken for infection [6], which is understandable given that cultures for some PG lesions return positive for concurrent infection [4,7]. For example, our patient’s course was complicated by actinomyces, MRSA, and *Klebsiella* infections.

A delay in diagnosing PG can lead to disease progression, exacerbated by aggressive debridement or other surgical intervention, particularly in patients exhibiting pathergy. While many cases of HN PG occur alongside lesions in typical sites such as the lower extremities [7], others remain isolated to the neck and head [8]. Despite its rarity, PG should be considered in patients presenting with ulcerative lesions in the head and neck, especially those with pre-existing systemic disease. Earlier diagnosis holds the potential to mitigate future morbidity associated with HN PG.

While the pathophysiology of PG has remained somewhat elusive, it is thought to stem from an immunological response to injury [4] where neutrophil dysfunction, abnormal inflammation, and genetic mutations play key roles in its clinical manifestations [1]. Biopsies of PG lesions are characterized by inflammatory neutrophilic dermal infiltrates, signifying neutrophilic dermatosis at its pathophysiology center. Abnormal neutrophil trafficking and dysregulated integrin signaling have also been described [3]. In addition, elevated levels of CD3+ T cells, macrophages, and interleukin 8 (IL-8) are found in PG lesions, aligning with their strong association with other inflammatory processes such as IBD and rheumatoid arthritis [1]. While our patient has no evidence of IBD, her positive ANA titer and history of PSC suggest inflammatory etiologies [3]. Furthermore, genetic diseases of immunity, including PAPA (Pyogenic Arthritis, Pyoderma Gangrenosum, and Acne) Syndrome, chronic granulomatous disease, and leukocyte adhesion deficiency, have been associated with PG, further complicating its pathogenesis [3].

PG is notoriously difficult to treat with no standardized national or international guidelines [8,9]. Nonetheless, management typically revolves around wound care, pain control, and reduction of inflammatory response [3]. Topical or intralesional corticosteroids suffice for small lesions (less than 2 cm^2^), while severe cases necessitate aggressive systemic therapy beginning with first-line corticosteroids and cyclosporine [10,11]. More recently, biological therapies such as tumor necrosis factor (TNF) blockers like infliximab and adalimumab have shown promising results, particularly in steroid-resistant cases such as our patient. Infliximab has been the most studied, and the only agent to be tested in a randomized controlled trial. This retrospective study determined that infliximab provided a definite cure of PG 22 out of 24 times (92%) and adalimumab, another TNF blocker, provided a definite cure 10 out of 10 times it was used. In contrast, oral corticosteroids were used first-line in almost 75% of patients but only provided a definite cure in 38% of the cases [12]. In our case, oral prednisone was not a cure or management for her chronic disease, despite playing a key role in her acute flares. Long-term corticosteroid therapy is not ideal due to the systemic side effects, including decreased bone density [13], and similarly, our patient's course was complicated by spinal compression fractures. She had the best response to infliximab but unfortunately could not maintain this treatment due to its cost. Conversely, other studies have reported PG anecdotal success [11].

Moreover, wound debridement is typically discouraged in PG due to the risk of pathergy, as demonstrated by the development of PG at post-surgical sites [4]. However, our patient received two debridements in the setting of a concomitant bacterial infection. This decision was made despite the risk of pathergy, which is potentiated if performed without adequate immunosuppression [14,15]. Our goal was to establish a fresh wound bed and promote re-epithelization as the wound was unlikely to heal otherwise. A systematic review of 161 PG patients who underwent surgical treatment found that treatment was successful in 139 cases (86%). However, there were 18 (11%) treatment failures, with the majority of these occurring in procedures that lacked adequate immunosuppression. Surgical approaches included a range of techniques including split-thickness skin grafts, negative pressure wound therapy, flap or dermal substitutes, punch grafts, and xenografts. Of note, none of the patients in this review exhibited pathergy [14]. This underscores the potential for surgical intervention in appropriately immunosuppressed patients, including our case, despite the major focus on medical management for PG.

As aforementioned, the surgical management of PG presents a complex therapeutic challenge often necessitating a multimodal approach including immunosuppression to achieve favorable outcomes, particularly in refractory cases or those with severe sequelae. The utilization of skin substitutes in treating pyoderma gangrenosum (PG) has been the subject of several studies aiming to improve wound healing and alleviate the debilitating effects of this ulcerative skin condition. Various approaches have been explored, including the use of biological skin substitutes, synthetic matrices, and autologous skin grafts. In a 2022 study by Nishimura et al., a treatment strategy for pyoderma gangrenosum involving skin grafting was discussed when combined with immunosuppressive drugs [16]. This study shows the evolution of a similar idea from a 1999 study by Cliff et al. [8]. They report on the use of split skin grafts in treating pyoderma gangrenosum, presenting findings from four cases [8]. Both studies explore the use of skin grafting in the treatment of pyoderma gangrenosum, a rare inflammatory skin condition. Nishimura et al. discussed a treatment strategy combining skin grafting with immunosuppressive drugs, suggesting a potentially more comprehensive approach to managing the condition [16]. In a case report by Toyozawa et al. [17], biologic skin substitutes such as acellular dermal matrices were found to promote wound closure and reduce recurrence rates in PG patients. Similarly, Pichler et al.’s systemic review of surgical treatment of PG with negative pressure wound therapy or skin grafting reported improved wound healing and reduced PG cases with these treatments [18]. While these studies collectively underscore the promise of skin substitutes in PG management, further research is warranted to optimize their efficacy, address potential complications, and refine treatment protocols for better clinical outcomes. As such, our study will contribute to a developing field assessing the durability and viability of adjunctive therapies in the management of PG.

## Conclusions

Our case of PG presents a unique challenge given the unusual location in the head and neck and the severity of its symptoms. While PG most commonly occurs on the lower extremities followed by breasts, abdomen, and other localizations, our patient's atypical presentation underscores the complexity of her condition. Additionally, size was a factor complicating our patient's treatment. In many studies of HN PG, the median ulcer size was less than 5 cm. However, our patient’s lesions were significantly larger and adequate control had not been achieved even after attempting multiple therapies. In conclusion, PG is a rare inflammatory disease that is difficult to diagnose and very challenging to treat. Its variable presentation as well as nonspecific laboratory and histologic findings can delay accurate diagnosis. Although involvement of the head and neck is rare, it is important to consider PG on the differential for patients presenting with non-healing lesions in this critical anatomic area.
